# In-vivo T2 mapping of atherosclerotic plaques in carotid arteries

**DOI:** 10.1186/1532-429X-14-S1-P134

**Published:** 2012-02-01

**Authors:** Luca Biasiolli, Alistair C Lindsay, Robin P Choudhury, Matthew D Robson

**Affiliations:** 1 Cardiovascular Medicine, University of Oxford, Oxford, UK; 2 Royal Brompton Hospital, London, UK

## Summary

The purpose of this study was to measure the T2 relaxation times of carotid atherosclerotic plaque components in-vivo at 3T and show the potential application of T2 mapping for plaque segmentation.

## Background

Clinical studies that have measured plaque T2 times were mostly performed ex-vivo using a small number of plaques excised from different arterial locations and imaged at different field strength using a limited number of TEs. The Table shows that T2 measured in the lipid-rich necrotic core (LRNC) was consistently shorter than T2 in fibrous tissue or in normal media, which showed similar values. The only two studies of carotid plaques showed a comparable T2 range for LRNC and fibrous tissue regardless of field strength difference.

## Methods

12 patients with stable atherosclerosis (9 males, 72±11 years) were imaged on a 3T scanner (Siemens TIM Trio). Ethics approval from local board was obtained and subjects gave informed consent. A multiple-Spin-Echo (multi-SE) sequence (Spin-Echo_Multi-Contrast or SE_MC) with low SAR pulses acquired black-blood cross-sectional images of carotid arteries using a 4-channel surface coil and cardiac gating (TE=25.8-38.7-51.6-64.5-77.4-90.3-103.2ms, TR=2R-R, FOV=160×128mm2, matrix-size=320×256, slice-thickness=2mm, partial-Fourier=5/8). T2 was estimated for every voxel of the carotid wall by fitting a mono-exponential decay curve to the signal intensities at 7 TEs using non-linear least-squares regression. Using a semi-automated method based on Bayes classifiers, T2 maps of carotid arteries were segmented in 4 tissue types: calcification; LRNC; fibrous tissue and normal media; intra-plaque haemorrhage. Histological validation was not available. AHA plaque classification was performed by two blinded reviewers on multi-contrast images acquired separately.

## Results

**Figure 1:**
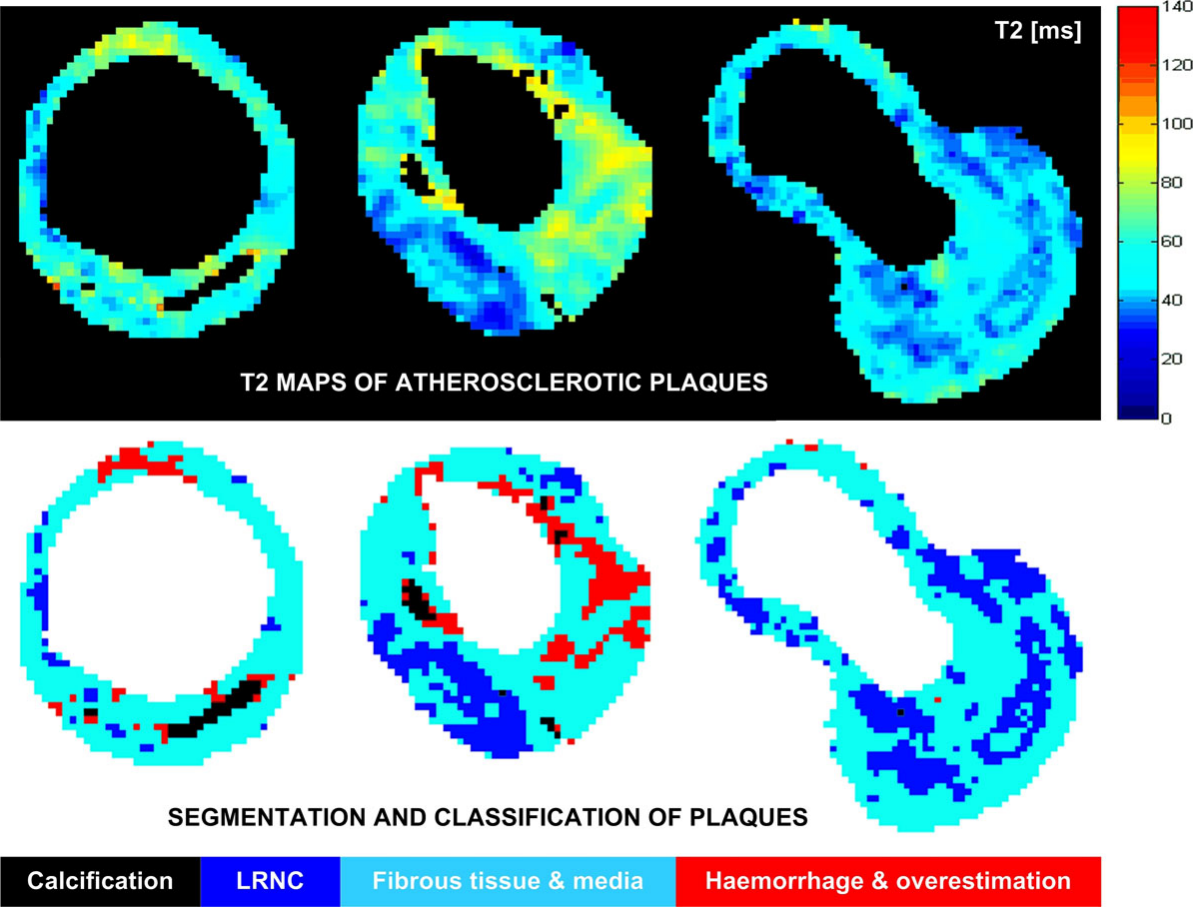
Cross-sectional T2 maps of carotid arteries with atherosclerotic plaques and relative classification of main plaque components. The colour scale indicates T2 relaxation times in ms with blue corresponding to LRNC (T2<40ms), cyan to normal media or fibrous tissue (T2=40-70ms) and red to haemorrhage (long T2). Yellow represents T2 values that may be caused by incorrect overestimation or by partial volume averaging in the presence of haemorrhage. Calcification has very low proton density that produces insufficient SNR and appears black on the T2 maps. Using a semi-automated method, T2 maps were segmented into 4 tissue classes represented by the different colours at the bottom.

23 carotid arteries presented visible lesions graded using the MRI-modified AHA scheme: 10 type III, 7 type IV-V, 2 type VI, 2 type VII and 2 type VIII plaques. From the T2 map segmentation of these arteries (Figure 1), 3438 voxels were classified as LRNC with T2=36±5ms and 10291 voxels as fibrous tissue or normal media with T2=55±9ms (Table 1). Due to low proton density, calcification produced insufficient SNR and T2 could not be measured. 1212 voxels were classified as haemorrhage with T2=89±20ms (some were incorrectly included due to T2 overestimation).

**Table 1:** T2 measurements [ms] of arterial wall and plaque tissues

Field strength	Study	Number of TEs	Number of plaques	Location	LRNC [ms]	Fibrous tissue [ms]	Normal media [ms]	References
1.5 T	ex-vivo	10	8	various	55 ± 3	79 ± 4	81 ± 3	*Toussaint et al.* (*1995*)
						
4.7 T					50 ± 3	63 ± 1	65 ± 2	
						
9.4 T		20			20 ± 3	30 ± 2	30 ± 3	

1.5 T	in-vivo	2	7	carotid	28 ± 6	51 ± 10	48 ± 7	*Toussaint et al.* (*1996*)
						
	ex-vivo				31 ± 5	51 ± 9	52 ± 7	

3 T	ex-vivo	7	14	aorta/iliac	54 ± 3	89 ± 6	76 ± 9	*Raynaud et al.* (*1998*)

9.4 T	ex-vivo	7	3	carotid	35 - 49	48 - 60	72 - 76	*Morrisett et al.* (*2003*)

4.7 T	ex-vivo	4	7	coronary	31 ± 7	55 ± 11	50 ± 10	*Sun et al.* (*2008*)

**3 T**	**in-vivo**	**7**	**23**	**carotid**	**36 ± 5**	**55 ± 9**		**this study**

*Toussaint et al.* (*1995*)*.* T2-weighted contrast for NMR characterization of human atherosclerosis. Arteriosclerosis Thrombosis and Vascular Biology, 15(10)1533-1542. *Toussaint et al.* (*1996*)*.* Magnetic Resonance Images Lipid, Fibrous, Calcified, Hemorrhagic, and Thrombotic Components of Human Atherosclerosis In Vivo. Circulation, 94(5)932-938. *Raynaud et al.* (*1998*)*.* Characterization of atherosclerotic plaque components by high resolution quantitative MR and US imaging. Journal of Magnetic Resonance Imaging, 8(3)622-629. *Morrisett et al.* (*2003*)*.* Discrimination of components in atherosclerotic plaques from human carotid endarterectomy specimens by magnetic resonance imaging ex vivo. Magnetic Resonance Imaging, 21(5)465-474. *Sun et al.* (*2008*)*.* Automatic plaque characterization employing quantitative and multicontrast MRI. Magnetic Resonance in Medicine, 59(1)174-180.

## Conclusions

This study showed the potential of in-vivo T2 mapping for atherosclerotic plaque characterization using Multi-SE in carotid imaging. T2 relaxation times measured in-vivo for LRNC and fibrous tissue or media at 3T were consistent with the range of values reported in literature for carotid plaques.

## Funding

This study was funded by EPSRC.

